# H_2_S suppresses indoleamine 2, 3-dioxygenase 1 and exhibits immunotherapeutic efficacy in murine hepatocellular carcinoma

**DOI:** 10.1186/s13046-019-1083-5

**Published:** 2019-02-18

**Authors:** Dan Yang, Tianqi Li, Yinlong Li, Shengnan Zhang, Weirui Li, Heng Liang, Zikang Xing, Lisha Du, Jinchao He, Chunxiang Kuang, Qing Yang

**Affiliations:** 1 State Key Laboratory of Genetic Engineering, Department of Biochemistry, School of Life Sciences, Fudan University, Songhu Road 2005, Shanghai, 200438 China; 2 Department of Chemistry, Tongji University, Siping Road 1239, Shanghai, 200092 China

**Keywords:** Cancer immunotherapy, Hydrogen sulfide, H_2_S/NO crosstalk, Indoleamine 2, 3 -dioxygenase 1, NF-κB and STAT3 signal pathways

## Abstract

**Background:**

Over-expression and over-activation of immunosuppressive enzyme indoleamine 2, 3 -dioxygenase 1 (IDO1) is a key mechanism of cancer immune escape. However, the regulation of IDO1 has not been fully studied. The relation between hydrogen sulfide (H_2_S) and IDO1 is unclear.

**Methods:**

The influences of endogenous and exogenous H_2_S on the expression of IDO1, iNOS and NF-κB and STAT3 signaling proteins were investigated using qPCR or western blot, and the production of nitric oxide (NO) was analyzed by nitrate/nitrite assay in *Cse*^*−/−*^ mice and MCF-7 and SGC-7901 cells. The effect of H_2_S on IDO1 activity was investigated by HPLC and in-vitro enzymatic assay. The effect of H_2_S on tryptophan metabolism was tested by luciferase reporter assay in MCF-7 and SGC-7901 cells. The correlation between H_2_S-generating enzyme CSE and IDO1 was investigated by immunostaining and heatmaps analysis in clinical specimens and tissue arrays of hepatocellular carcinoma (HCC) patients. The immunotherapeutic effects of H_2_S on H22 HCC-bearing mice were investigated.

**Results:**

Using *Cse*^*−/−*^ mice, we found that H_2_S deficiency increased IDO1 expression and activity, stimulated NF-κB and STAT3 pathways and decreased the expression of NO-generating enzyme *Inos*. Using IDO1-expressing MCF-7 and SGC-7901 cells, we found that exogenous H_2_S inhibited IDO1 expression by blocking STAT3 and NF-κB pathways, and decreased IDO1 activity via H_2_S/NO crosstalk, and combinedly decreased the tryptophan metabolism. The negative correlation between H_2_S-generating enzyme CSE and IDO1 was further validated in clinical specimens and tissue arrays of HCC patients. Additionally, H_2_S donors effectively restricted the tumor development in H22 HCC-bearing mice via downregulating IDO1 expression, inducing T-effector cells and inhibiting MDSCs.

**Conclusions:**

Thus, H_2_S, as a novel negative regulator of IDO1, shows encouraging antitumor immunotherapeutic effects and represents a novel therapeutic target in cancer therapy.

**Electronic supplementary material:**

The online version of this article (10.1186/s13046-019-1083-5) contains supplementary material, which is available to authorized users.

## Background

Indoleamine 2,3-dioxygenase 1 (IDO1), a ubiquitous monomeric heme-containing enzyme, is the first rate-limiting enzyme in the kynurenine pathway (KP) that catabolizes tryptophan (Trp) into kynurenine [1]. Over-expression and over-activation of IDO1 in tumors and antigen-presenting cells play important roles in tumor-induced tolerance and suppression of the immune system by depleting the essential amino acid tryptophan and producing toxic tryptophan metabolites [2–4]. As a result, the activation of effector T cells (Teff) is inhibited, the differentiation and activation of Foxp3^+^ regulatory T cells (Tregs) is enhanced, and the recruitment of myeloid-derived suppressor cells (MDSCs) is increased. Moreover, the tryptophan metabolites L-kynurenine (L-Kyn) [5], 6-formylindolcarbazole [6] and kynurenic acid [7] are aromatic hydrocarbon receptor (AhR) ligands with strong immunosuppressive potency. Consistently, pharmacological inhibition of IDO1 reverses the immune suppression in tumor microenvironments and improves the efficacy of therapeutic vaccination, chemotherapy or radiation therapy [8–10]. Thus, inhibition of IDO1 has emerged as an attractive strategy for cancer immunotherapy.

Given the important functions of IDO1, its expression must be under tight regulation. IDO1 can be induced by specific inflammatory stimuli, such as IFN-gamma (IFN-γ) or lipopolysaccharide (LPS) [11, 12]. LPS and IFN-γ up-regulate IDO1 expression by activating the nuclear factor-κB (NF-κB) pathway [13, 14]. IDO1 is also constitutively expressed via an autocrine AhR-IL-6-STAT3 signaling loop in IDO1-positive cancer cells [15]. Recent studies show that nitric oxide (NO), a gaseous signaling molecule produced by inducible nitric oxide synthase (iNOS), suppresses IFN-γ-induced IDO1 expression [16]. Furthermore, NO builds the IDO1-NO-Trp complex to inhibit IDO1 activity in a reversible manner [17]. In addition, NO induces IDO1 degradation and antagonizes IDO1-dependent tolerogenesis by mediating the binding of the suppressor of cytokine signaling 3 (SOCS3) at a specific phosphotyrosine [18, 19]. It suggests that NO is a modulator for IDO1 function and it would be of great interest to investigate whether and how IDO1 is modulated by gaseous signaling molecules beyond NO.

H_2_S is another gaseous signaling molecule [20], and it is involved in a wide range of cellular functions including vasorelaxation, angiogenesis, cellular energy production, neuromodulation, and cytoprotection [21]. In mammals, H_2_S is produced from the metabolism of L-cysteine and homocysteine by catalytic enzymes, cystathionine γ-lyase (CSE aka CTH, CGL), cystathionine β-synthase (CBS) and 3-mercaptopyruvate sulfurtransferase (3-MST) [22]. CSE is distributed in a wider range of tissues than CBS^22^. H_2_S exhibits anti-cancer activity by inducing uncontrollable intracellular acidification [23], disturbing energy metabolism [24] and inhibiting angiogenesis [25]. Additionally, H_2_S relieves inflammatory reaction by interfering with the ability of neutrophils and inducing neutrophils apoptosis [26]. The inflammatory or immune reaction caused by the infiltration of some immune cells such as granulocytes, lymphocytes, and macrophages plays an important role in the development of tumors. However, whether and how H_2_S can exert anti-tumor effects through immune regulation is still unknown.

Here, we perform a comprehensive investigation on the effects of H_2_S on the expression and activity of IDO1 and tryptophan metabolism. We find that H_2_S downregulates IDO1 expression by blocking NF-κB and STAT3 pathways, and inhibits IDO1 activity via H_2_S/NO crosstalk. H_2_S suppresses IDO1 expression and exhibits immunotherapeutic efficacy in H22 hepatocellular carcinoma (HCC)-bearing mice. Our data elucidate the role of H_2_S in the regulation of IDO1 and provide a novel strategy to target H_2_S for cancer immunotherapy.

## Materials and methods

### Data reporting

No statistical methods were used to predetermine sample size. For animal studies, the investigators were not blinded to allocation during experiments and outcome assessment. Immunohistochemically stained tissue sections were reviewed and scored separately by two pathologists blinded to the clinical parameters.

### Animal model and treatments

Female, 8-week-old, wild-type C57BL/6 and female, four- to 6-week-old wild-type Kunming mice were purchased from SLAC Experimental Animal Center, Shanghai, China. Female, 8-week-old, *Cse* knockout (*Cse*^*−/−*^) C57BL/6 mice were provided by the School of Basic Medical Sciences, Fudan University (Shanghai, China). The experimental procedures were approved by the Animal Ethics Committee of Fudan University, experiments were performed in compliance with ARRIVE guidelines. Detailed information on H22 HCC-bearing mouse construction and treatments can be found in Additional file 1.

### Human samples

Clinical specimens of HCC patients (9 cases) and tissue arrays of HCC patients (158 cases) and MHCC patients (158 cases) were obtained from Zhongshan Hospital Affiliated to Fudan University. All tissues were collected with the donor being informed completely and with their consent. The procedures were approved by the Medical Ethics Committee of Fudan University.

### Cell culture and transfection

Human breast cancer cells (MCF-7), human gastric carcinoma cells (SGC-7901) and murine H22 hepatocellular carcinoma cells were bought from the cell bank of the Chinese academy of sciences (Shanghai, China). More information can be found in Additional file 1.

### Quantitative real-time PCR (qPCR) and western blot analysis

Total RNA was isolated using Trizol (Invitrogen, CA, USA) following the manufacturer’s guidelines. Reverse transcription and qPCR analysis were performed as previously described [9]. qPCR analysis was performed in quadruplicate for each sample with specific primers (Additional file 2: Table S1). Detailed information of western blot and the primary antibodies used in the experiment can be found in Additional file 1.

### IDO1 inhibition assay and IC_50_ assay

The assays were performed as described previously [51].

### Detection of NO production

NO production was measured by a Nitrate/Nitrite Assay Kit (Beyotime, China) following the manufacturer’s guidelines. Detailed information can be found in Additional file 1.

### High-performance liquid chromatography (HPLC) analysis

The IDO1 activities of mouse serum and cell supernatant were analyzed by an Agilent 1260 series HPLC system (Agilent Corp., USA). More information can be found in Additional file 1.

### Expression vector construction

The three recombinant vectors used in this study were pcDNA-3.1(+)-CSE, px459-cas9-CSE and pGL3- Promoter-DRE. The human CSE sequences that were derived from amplifying the cDNA from SGC-7901 genomes with special primers containing EcoRI (sense, S) and XhoI (antisense, R) restriction enzyme site sequences (Additional file 2: Table S2) were subcloned into a pcDNA-3.1(+) vector to construct a pcDNA-3.1(+)-CSE recombinant vector. The px459-cas9-CSE vector targeting human CSE was constructed with special gRNAs sequences (Additional file 2: Table S2). And the six DRE arrays (sense: TCGCGTG and antisense: AGCGCAC) containing KpnI (sense, S) and BglII (antisense, R) restriction enzyme site sequences (Additional file 2: Table S2) were subcloned into a pGL3-promoter vector yielding a pGL3-Promoter-DRE vector with a luciferase reporter.

### Luciferase reporter assays

MCF-7 and SGC-7901 cells were transiently transfected with pGL3-Promoter-DRE vector and pSV-β-Galactosidase control vector for 12 h, then treated with different concentration of H_2_S donors for 24 h. These cells were lysated and analyzed by a luciferase assay using the luciferase assay kit (Promega, WI, USA) according to the manufacturer’s instructions.

### Immunostaining and H&E staining

Detailed procedure and antibody information can be found in Additional file 1.

### Analyzing the correlations between IDO1 expression and CSE expression, CD11b^+^ myeloid cell number or CD8^+^ T cell number

Detailed procedure of Spearman rank correlation analysis can be found in Additional file 1.

### Statistical analysis

Data are expressed as mean ± SD or SEM. One-way analysis of variance (ANOVA) followed by Dunnett’s post hoc test was used to compare several treatment groups with one control group. Student’s t-test was used to determine the difference between two groups. Prism 6 software (GraphPad Software) was used to create the graphs and implement the statistical analysis. The western blot was repeated at least three times. Immunostaining intensity, cell number and cell area analysis were used by Image J software. Correlations between IDO1 expression and CSE expression, CD11b^+^ myeloid cell number or CD8^+^ T cell number were analyzed by Spearman rank correlation (GraphPad Software or R). Heatmaps analysis for correlations between IDO1 expression and CSE expression, CD11b^+^ myeloid cell number or CD8^+^ T cell number were performed by R statistical software. Significance values were set at *p < 0.05, **p < 0.01 and ***p < 0.001.

## Results

### H_2_S deficiency increased the expression and activity of IDO1 in *Cse*^*−/−*^ mice

**Fig. 1:**
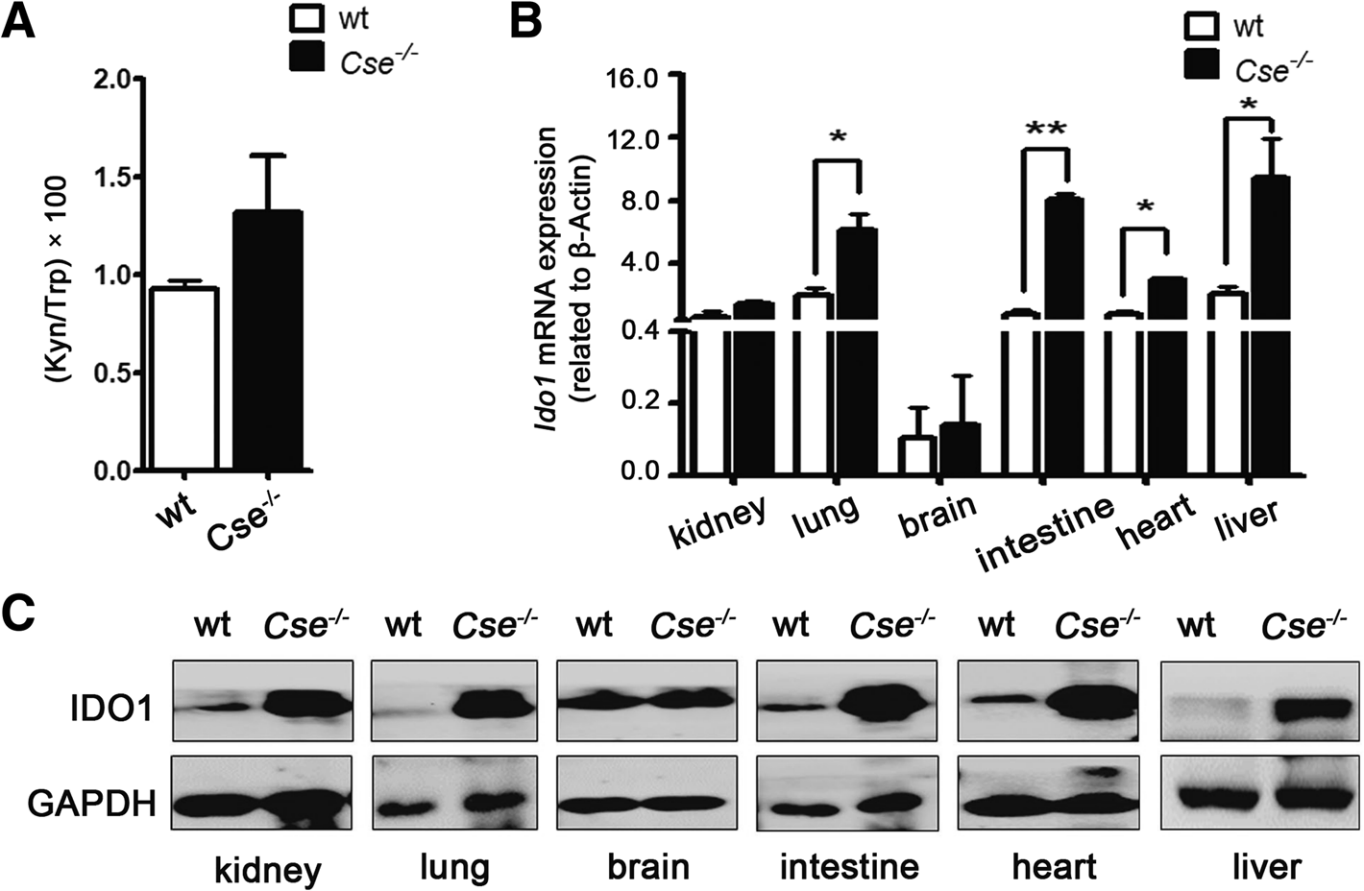
H_2_S deficiency increased the expression and activity of IDO1 in *Cse*^*−/−*^ mice. **a** HPLC analysis of Kyn/Trp ratios in wild-type (wt) mice and *Cse*^*−/−*^ mice, *N* = 6. **b**, **c** Analysis of IDO1 expression in different tissues of wt mice and *Cse*^*−/−*^ mice. Total RNA and protein were extracted from different tissues of wt mice and *Cse*^*−/−*^ mice. mRNA expression of *Ido1* was analyzed by qPCR, values from three independent experiments are presented as the mean ± SEM, *p < 0.05, **p < 0.01, Student’s t test. Protein expression of IDO1 was analyzed by western blot

NO has been known to suppress IDO1 expression and inhibit IDO1 activity in a reversible manner [16, 17]. Additionally, H_2_S has a correlation with NO [27–29]. However, the relation between H_2_S and IDO1 is unclear. To test if H_2_S regulates IDO1, we measured the serum IDO1 activity and expression profile in different tissues of *Cse*^*−/−*^ mice, a genetic mouse model that has a lower level of endogenous H_2_S than that of wild-type mice [30]. Serum IDO1 activity, described as the Kyn/Trp ratio, in *Cse*^*−/−*^ mice was higher than that in wild-type mice **(**Fig. 1a). Both the mRNA and protein levels of IDO1 in the kidney, lung, intestine, heart, and liver of *Cse*^*−/−*^ mice were higher than that in wild-type mice except that in the brain (Fig. 1b and c). This finding showed that in-vivo H_2_S deficiency increased the expression and activity of IDO1, implying the potential modulation of H_2_S on IDO1.

### H_2_S downregulated IDO1 expression by blocking NF-κB and STAT3 pathways

**Fig. 2:**
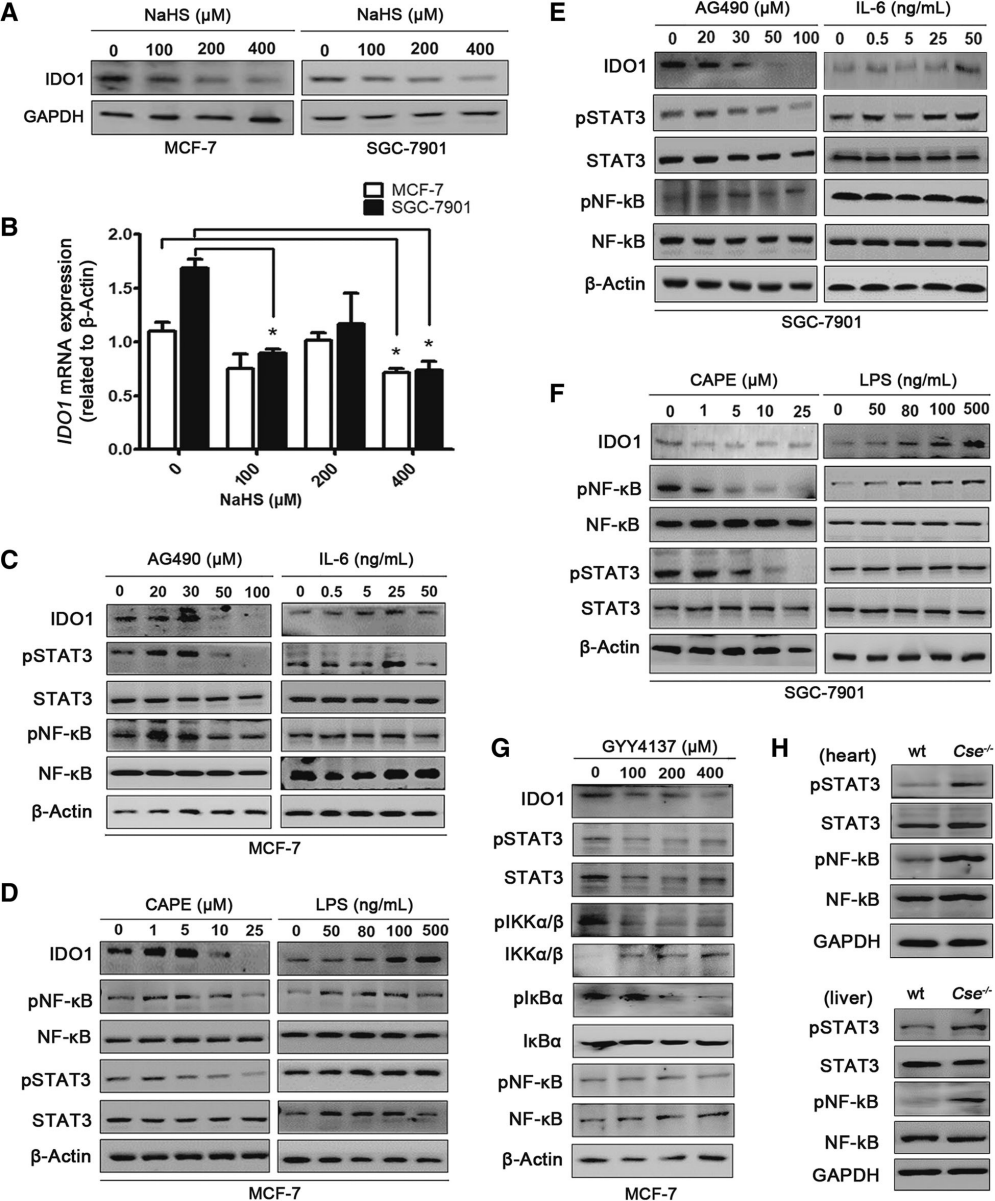
H_2_S downregulated IDO1 expression by blocking NF-κB and STAT3 pathways. **a**, **b** The H_2_S donor downregulated IDO1 expression in MCF-7 and SGC-7901 cells. MCF-7 and SGC-7901 cells were treated with different concentrations (0–400 μM) of NaHS for 24 h, protein and mRNA expression of IDO1 were detected by western blot and qPCR, respectively. Statistical significance was determined by one-way ANOVA followed by Dunnett’s test. *p < 0.05, bars show the group mean ± SEM. **c-f** IDO1 expression was regulated by NF-κB and STAT3 pathways in MCF-7 and SGC-7901 cells. Total proteins were extracted from MCF-7 and SGC-7901 cells after being treated with AG490 (0–100 μM) for 24 h, IL-6 (0–50 ng/mL) for 48 h, CAPE (0–25 μM) for 48 h or LPS (0–500 ng/mL) for 24 h and analyzed by western blot. **g** The H_2_S donor downregulated IDO1 expression by blocking NF-κB and STAT3 pathways in MCF-7 cells. Total proteins were extracted from MCF-7 cells that were treated with different concentrations of GYY4137 for 24 h and were analyzed by western blot. **h** H_2_S deficiency increased the phosphorylation levels of NF-κB and STAT3 pathways in *Cse*^*−/−*^ mice. Total proteins were extracted from heart and liver tissues of wt mice and *Cse*^*−/−*^ mice, and proteins expression were determined by western blot

IDO1-expressing breast cancer cell line MCF-7 and gastric carcinoma cell line SGC-7901 were chosen as cell models to elucidate the potential role of H_2_S in the regulation of IDO1. It was found that the exposure to exogenous H_2_S donors (NaHS or GYY4137) led to the decrease in both the protein and mRNA expression of IDO1 in MCF-7 and SGC-7901 cells (Fig. 2a and b). Further, we constructed CSE over-expressing (CSE OE) MCF-7 and SGC-7901 cells and CSE knockout (CSE KO) SGC-7901 cells to analyze the IDO1 expression. IDO1 expression in CSE OE cells was decreased when compared to the control cells transfected with empty vector pcDNA-3.1(+) (Additional file 3: Figure S1A and S1B). IDO1 expression in CSE KO SGC-7901 cells was significantly upregulated when compared to the control cells transfected with empty vector px459-cas9 (Additional file 3: Figure S1C-E). This result indicated that both exogenous and endogenous H_2_S suppressed IDO1 expression.

Next, due to the well-established roles of STAT3 and NF-κB pathways in regulating IDO1 expression, we sought to demonstrate whether these two pathways mediate the suppression of H_2_S on IDO1 expression in MCF-7 and SGC-7901 cells. Tyrphostin B42 (AG490) and caffeic acid phenethyl ester **(**CAPE) were used to inhibit STAT3 and the NF-κB phosphorylation, respectively [15]. Interleukin-6 (IL-6) and LPS were used to induce the STAT3 and NF-κB phosphorylation, respectively [13, 31, 32]. In MCF-7 cells, inhibiting STAT3 phosphorylation with AG490 (50–100 μM) decreased the expression level of IDO1, and activating STAT3 phosphorylation with IL-6 (5–25 ng/mL) increased the expression level of IDO1 (Fig. 2c). Inhibition of NF-κB phosphorylation with CAPE (10–25 μM) decreased the expression level of IDO1 (Fig. 2d) while activation of NF-κB phosphorylation with LPS (80–100 ng/mL) increased the expression level of IDO1. These results were fully reproduced in another cell line (SGC-7901) (Fig. 2e and f). To demonstrate that H_2_S might regulate IDO1 expression via STAT3 and NF-κB pathways, we examined the effects of the H_2_S donor on both pathways and IDO1 expression. It was found that GYY4137 simultaneously down-regulated the expression of IDO1 and the phosphorylation levels of STAT3 and NF-κB in MCF-7 cells in a dose-dependent manner (Fig. 2g). Additionally, the phosphorylation levels of IKKα/β and IκB, the upstream kinases of NF-κB, were significantly decreased after GYY4137 treatment. This result showed that GYY4137 did affect the phosphorylation of NF-κB. In addition, we evaluated the phosphorylation levels of the STAT3 and NF-κB in heart and liver tissues in *Cse*^*−/−*^ mice and wild-type mice. The levels of pSTAT3 and pNF-κB in the heart and liver of *Cse*^*−/−*^ mice were higher than those in wild-type mice (Fig. 2h). Taken together, these data further demonstrated that H_2_S down-regulated IDO1 expression by blocking STAT3 and NF-κB pathways.

### H_2_S inhibited IDO1 activity via H_2_S/NO crosstalk, and NO rather than H_2_S was an IDO1 inhibitor

**Fig. 3:**
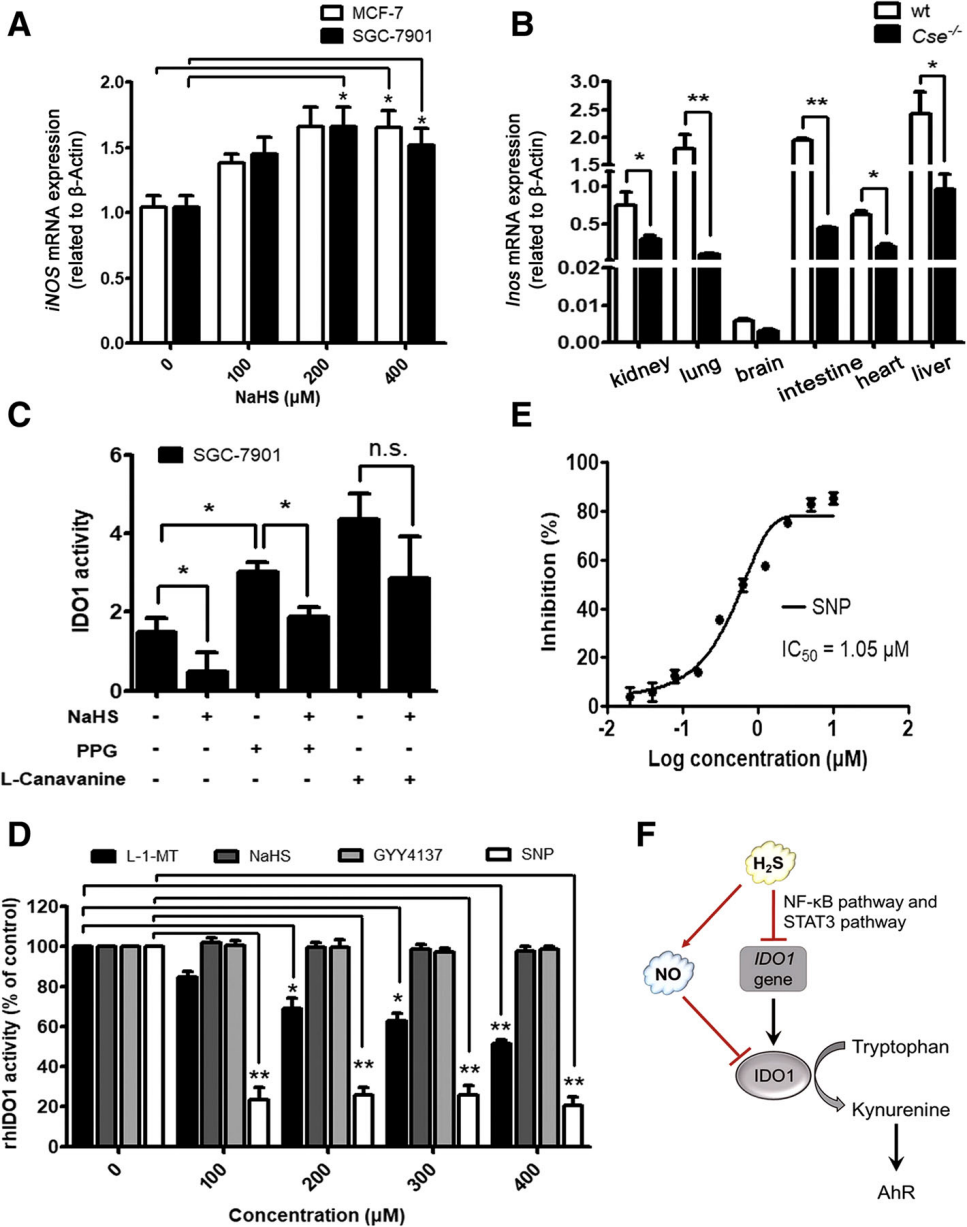
H_2_S inhibited IDO1 activity via H_2_S/NO crosstalk, and NO rather than H_2_S was an IDO1 inhibitor. **a** H_2_S donor upregulated the mRNA expression of *iNOS* in MCF-7 and SGC-7901 cells. Total RNA was extracted from the cells that were treated with different concentrations of NaHS for 24 h, and the mRNA expression levels of *iNOS* were analyzed by qPCR. **b** H_2_S deficiency downregulated the mRNA expression of *Inos* in *Cse*^*−/−*^ mice. Total RNA was extracted from different tissues of the wt mice and *Cse*^*−/−*^ mice, and the mRNA expression levels of *Inos* were analyzed by qPCR. **c** NO played an important role in the regulation of H_2_S on IDO1 activity. The cell supernatants were harvested, and kynurenine and tryptophan levels were measured by HPLC. **d**, **e** NO rather than H_2_S was a direct inhibitor of IDO1. Enzymatic assay of IDO1 was carried out with recombinant human IDO1 (rhIDO1) and different concentrations (0–400 μM) of H_2_S donors (NaHS, GYY4137), NO donor (SNP) and 1-L-MT. **f** Schematic diagram depicting the modulation of H_2_S on IDO1. All experiments repeated at least three times. Statistical significance was determined by Student’s t test (**b**) and one-way ANOVA followed by Dunnett’s test (**a**, **c** and **d**). *P < 0.05, **P < 0.01, n.s., no significant difference, bars show the group mean ± SEM

In the present study, we observed that H_2_S donor increased both the mRNA level of *iNOS* gene (Fig. 3a) and the activity of iNOS in a dose-dependent manner in MCF-7 and SGC-7901 cells (Additional file 3: Figure S2A). To further certify the relationship between H_2_S and NO, the mRNA levels of *Inos* in different tissues of *Cse*^*−/−*^ mice were detected and were found to be significantly lower than that in wild-type mice (Fig. 3b). In IDO1 and CSE co-expressing SGC-7901 cells, the treatment of NaHS (400 μM, 1 h) significantly decreased the activity of IDO1 (Fig. 3c) but increased the production of NO (Additional file 3: Figure S2B). Correspondingly, the inhibition of endogenous CSE with DL-propargylglycine (PPG, CSE inhibitor, 10 mM, 4 h) increased the activity of IDO1 (Fig. 3c) and decreased the production of NO (Additional file 3: Figure S2B). Moreover, the supplement of NaHS (400 μM, 1 h) after the pretreatment of PPG alone (CSE inhibitor, 10 mM, 4 h), significantly decreased the enhanced activity of IDO1 caused by PPG treatment. Similarly, the supplement of NaHS after the pretreatment of PPG alone remarkably reversed the decreased production of NO due to PPG treatment. This result indicated that the exogenous H_2_S could decrease the activity of IDO1 even when the endogenous formation of H_2_S was blocked. Interestingly, the inhibition of iNOS with L-canavanine (iNOS inhibitor, 200 μM, 2 h) decreased the production of NO and increased the activity of IDO1. However, the supplement of NaHS (400 μM, 1 h) after the pretreatment of L-canavanine alone, did not significantly changed the IDO1 activity and NO production caused by L-canavanine (Fig. 3c and Additional file 3: Figure S2B). These results suggested that NO played an important role in the regulation of H_2_S on IDO1 activity.

To further explore if H_2_S and NO inhibit the activity of IDO1, we purified the recombinant human IDO1 (rhIDO1) and carried out the in-vitro enzymatic assay. The inhibitory activity of exogenous H_2_S donors (NaHS, GYY4137) and NO donor (sodium nitroprusside, SNP) on IDO1 was evaluated, and the typical IDO1 inhibitor L-1-methyl-tryptophan (L-1-MT) was used as the positive control (Fig. 3d). The results showed that H_2_S donors (NaHS, GYY4137) had no IDO1 inhibitory activities, but the NO donor (SNP) showed potent inhibitory activity, and its inhibitory effect is even better than L-1-MT. 100 μM SNP could achieve 80% IDO1 inhibitory activity. Moreover, the IC_50_ value of SNP was calculated as low as 1.05 μM (Fig. 3e). Combined data demonstrated that NO rather than H_2_S was a direct inhibitor of IDO1, and H_2_S inhibited the activity of IDO1 via H_2_S/NO crosstalk.

Based on the inhibitory effects of H_2_S on IDO1 activity and expression, we investigated the effect of H_2_S on tryptophan metabolism. Herein, luciferase reporter assay of kynurenine, a tryptophan metabolite, was performed. NaHS and GYY4137 were supplemented to MCF-7 and SGC-7901 cells transfected with pGL3-Promoter-DRE vector, and the cell lysates were analyzed by luciferase reporter assay. The luciferase activity decreased in a dose-dependent manner upon exogenous H_2_S treatment, indicating that the concentration of kynurenine was decreased, and thus tryptophan metabolism was decreased (Additional file 3: Figure S3A and S3B). Kynurenine can activate the transcription of *CYP1A1* (cytochrome P4501A1) and *CYP1B1* (cytochrome P4501B1) which are the target genes of AhR-Ah receptor nuclear translocator (AhR-ARNT) [33, 34]. Therefore, we also investigated the influence of H_2_S on *CYP1A1* and *CYP1B1* expression. It was found that the mRNA expression levels of both *CYP1A1* and *CYP1B1* decreased significantly upon the treatment of GYY4137 of different concentrations in MCF-7 and SGC-7901 cells (Additional file 3: Figure S3C and S3D). Based on the data of luciferase reporter assay and *CYP1A1*/*CYP1B1* quantification, it was concluded that H_2_S was able to decrease tryptophan metabolism. Taken together, H_2_S down-regulated IDO1 expression, inhibited IDO1 activity, and decreased tryptophan metabolism.

### IDO1 expression was negatively correlated with CSE expression in clinical specimens and tissue arrays of HCC patients

**Fig. 4:**
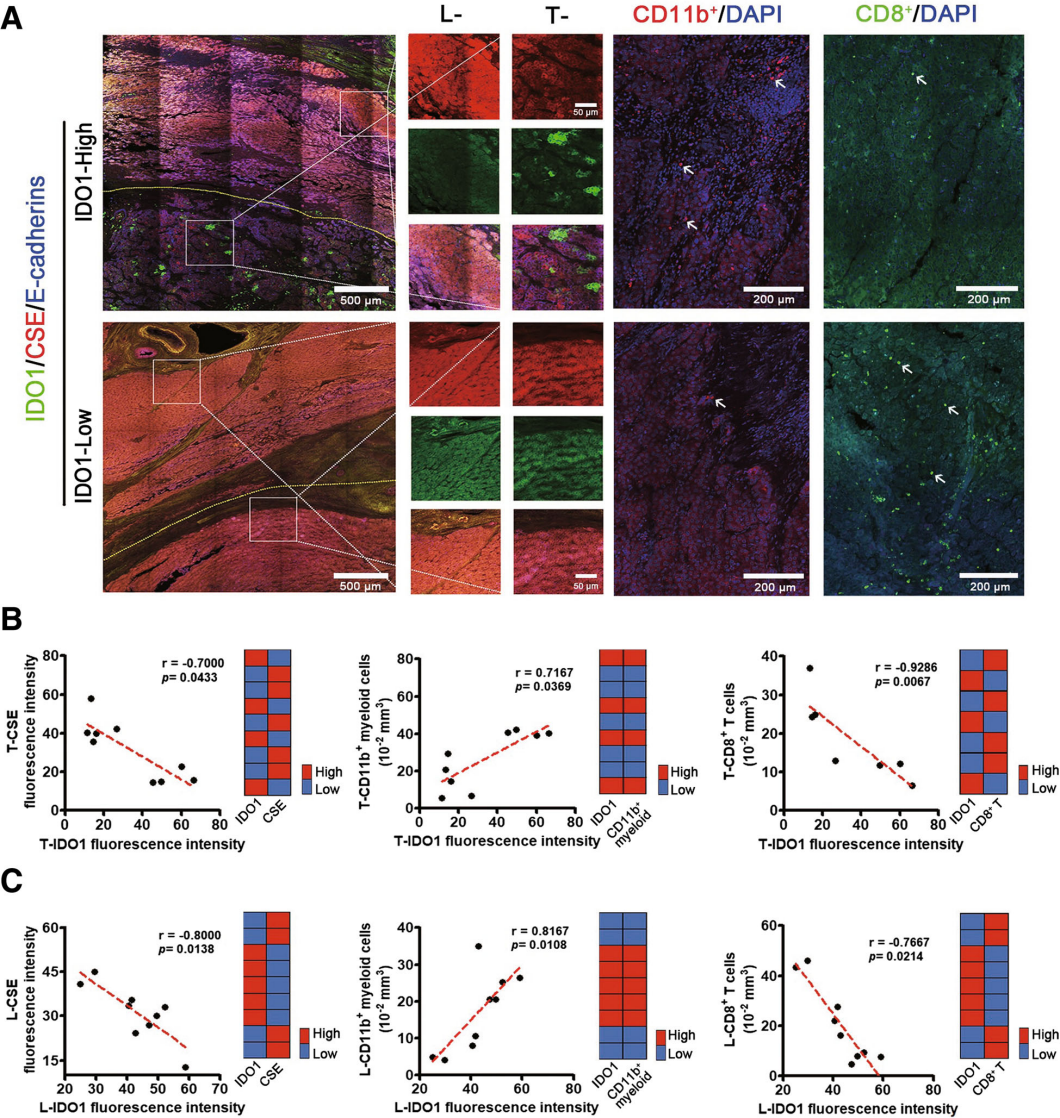
Correlations between IDO1 expression and CSE expression, CD11b^+^ myeloid cell number or CD8^+^ T cell number in HCC patients. **a** The representative immunostaining for IDO1, CSE, CD11b^+^ myeloid cells and CD8^+^ T cells (× 200 magnification) in the tumor (Tumor, T-) and adjacent non-neoplastic tissues (Liver, L-) of HCC patients. **b** Correlations between IDO1 expression (*N* = 9) and CSE expression (*N* = 9), CD11b^+^ myeloid (*N* = 9) cell number or CD8^+^ T (*N* = 7) cell number in the tumor (Tumor, T-) tissues of HCC patients. The left panel of each figure illustrates scatter gram with its Spearman correlation coefficient and *P* value. The right panel of each figure shows the heatmap. **c** Correlations between IDO1 expression and CSE expression, CD11b^+^ myeloid cell number or CD8^+^ T cell number in adjacent non-neoplastic tissues (Liver, L-) of HCC patients. *N* = 9. The left panel of each figure illustrates the scatter gram with its Spearman correlation coefficient and *P* value. The right panel of each figure shows the heatmap

**Fig. 5:**
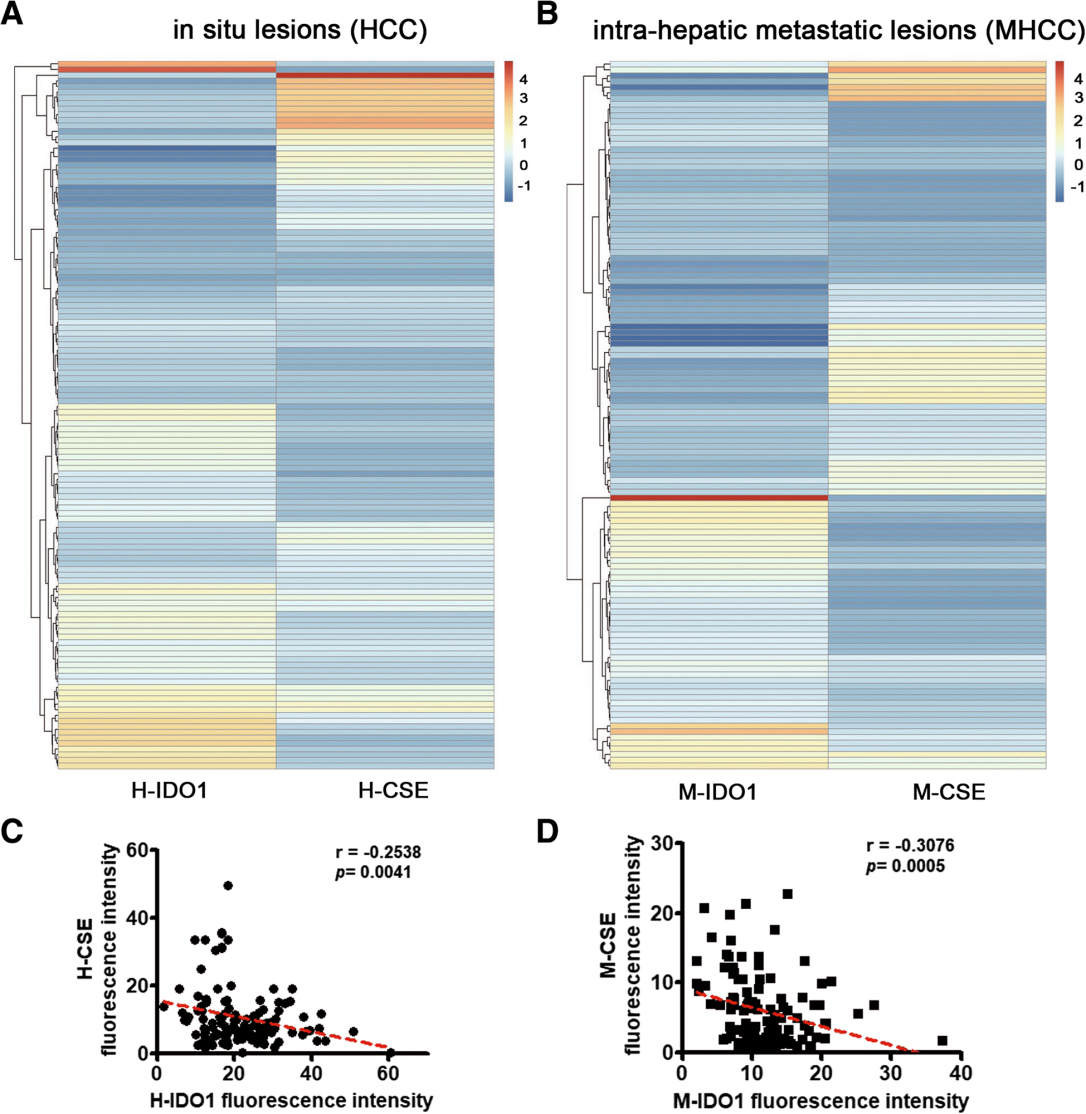
Correlation between IDO1 expression and CSE expression in tissue arrays of HCC and MHCC patients. **a**, **b** Heatmaps showing the correlation between IDO1 expression and CSE expression in tissue arrays of HCC and MHCC patients. **c**, **d** Scatter grams showing the correlation between IDO1 expression and CSE expression in tissue arrays of HCC and MHCC patients. H-: In situ lesions of HCC patients, *N* = 126 (valid values collected); M-: Intra-hepatic metastatic lesions of MHCC patients, *N* = 124 (valid values collected)

Given the correlation between H_2_S and IDO1 found in *Cse*^*−/−*^ mice, breast cancer cell line MCF-7 and gastric carcinoma cell line SGC-7901, it is necessary to find the universality of this correlation in human cancer samples. HCC is one of the leading causes of death in the world. Thus, the expression profiles of IDO1 and CSE in clinical specimens and tissue arrays of HCC patients were investigated. It was determined that IDO1 and CSE were expressed in both tumor tissues (Tumor, T-) and adjacent non-neoplastic tissues (Liver, L-) of nine HCC patients. In both IDO1-high and IDO1-low groups (definition standards see Additional file 1), the negative correlation between IDO1 expression and CSE expression in both T- and L- tissues was observed (Fig. 4a). The scatter gram of the data from nine HCC patients further confirmed this result (Fig. 4b and c). It has been well known that tumor IDO1 promotes immunosuppression by direct action on effector T cells and Tregs, and through recruitment, expansion and activation of MDSCs [4, 35]. Herein, CD11b^+^ myeloid cells were found to aggregate in IDO1 high-expressed (IDO1-high) but not in IDO1 low-expressed (IDO1-low) T- tissues, and CD11b^+^ myeloid cell number was positively correlated with IDO1 expression (Fig. 4a and b). However, CD8^+^ T cells were found to aggregate in IDO1-low but not in IDO1-high T- tissues, and CD8^+^ T cell number was negatively correlated with IDO1 expression (Fig. 4a and b). Similar results were obtained in L- tissues (Fig. 4c). Next, using the tissue arrays of HCC patients (158 cases) and metastatic hepatocellular carcinoma (MHCC) patients (158 cases) (Additional file 3: Figure S4), we further verified the negative relation between CSE expression and IDO1 expression by some statistical analysis and empirical tests. Although the expression levels of IDO1 in individual patients were quite different, the heatmap and scatter gram indicated that IDO1 expression was negatively correlated with CSE expression (Fig. 5). And this correlation was verified by the negative Spearmen correlation coefficient (Fig. 5c and d). Taken together, the negative relation between CSE expression and IDO1 expression, as well as the association of high IDO1 expression with immunosuppression was found to exist in HCC patient.

### H_2_S restricted tumor growth in H22 HCC-bearing mice

**Fig. 6:**
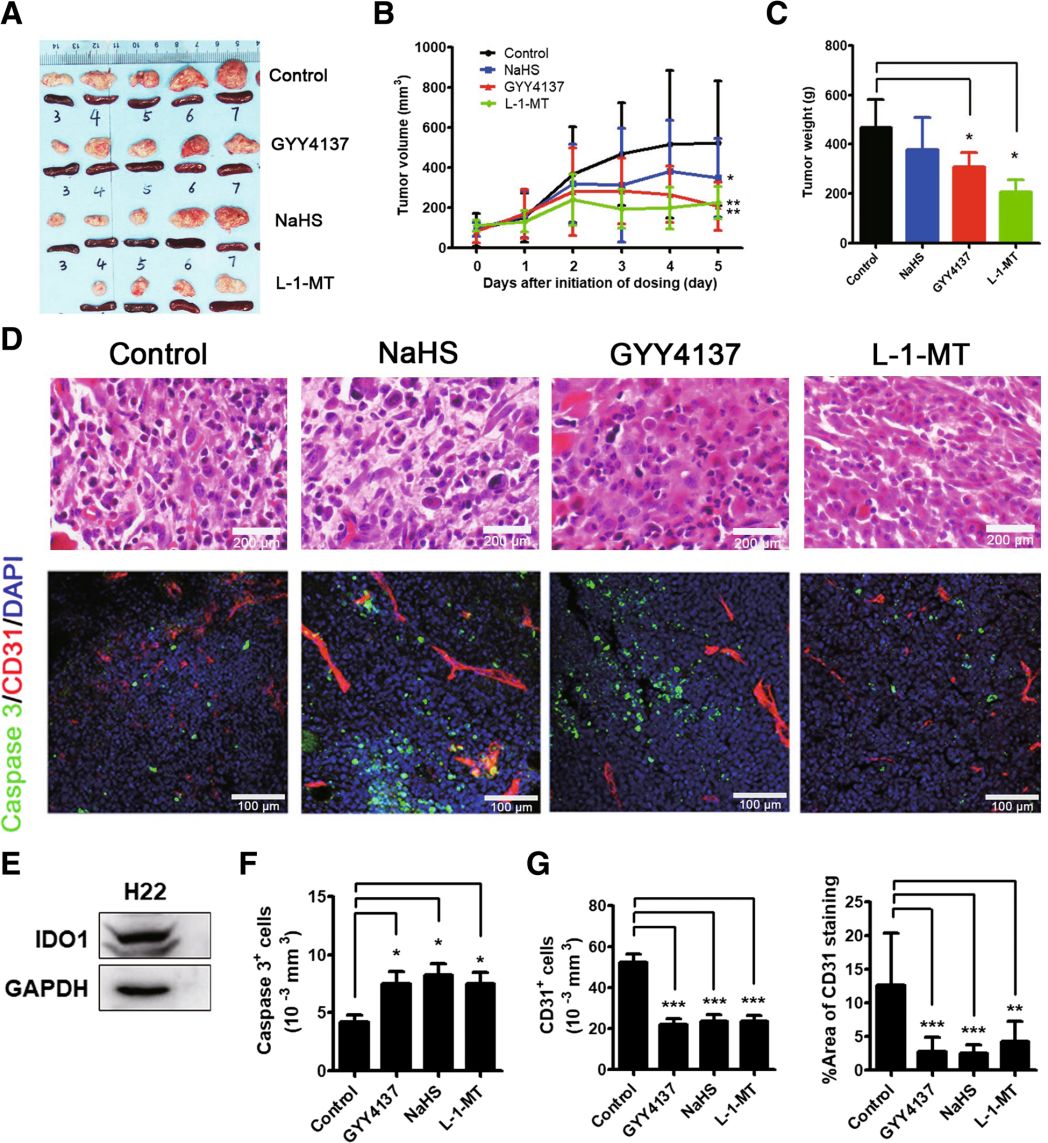
H_2_S restricted tumor growth in H22 HCC-bearing mice. **a** Representative images of tumors and spleens from four groups of mice (L-1-MT group, *N* = 4, other groups, *N* = 9) after being euthanized at the endpoint of study. Tumors were arrayed according to tumor size from small to large. **b** H22 tumor growth curve of different groups of mice. **c** Tumor weight measured from four groups of mice after being euthanized at endpoint of study. **d** H&E staining of H22 tumors from four groups of mice (× 400 magnification). Representative immunostaining of Caspase 3^+^ and CD31^+^ in tumors from four groups of mice (× 200 magnification). **e** The IDO1 expression in H22 cells demonstrated by western blot. **f**, **g** Quantification of Caspase 3 and CD31 positive cells of H22 tumors from four groups of mice. L-1-MT group, *N* = 4, other groups, *N* = 9. Statistical significance was determined by one-way ANOVA followed by Dunnett’s test. *P < 0.05, **P < 0.01, ***P < 0.001, bars show the group mean ± SD

Based on the correlation between H_2_S and IDO1, and the role of IDO1 in tumor immunosuppression, it is possible to assume that H_2_S may have immunotherapeutic activity. Using H22 HCC-bearing mice, we carried out the in-vivo antitumor assay of H_2_S donors NaHS and GYY4137, and a typical IDO1 inhibitor L-1-MT. H22 cells are IDO1 expressing (Fig. 6e) and highly tumorigenic [36] hepatocellular carcinoma cells (Fig. 6d). At the end of treatment, GYY4137 (60.1% reduction), L-1-MT (56.7% reduction) and NaHS (33.3% reduction) significantly restricted tumor growth (Fig. 6a and b). Similarly, both L-1-MT and H_2_S donors reduced tumor weight at the end of study (Fig. 6c). In addition, the mRNA expression of *Inos* and the production of NO in tumors of H_2_S treated mice were higher than that in the control mice. However, there was no difference in *Inos* mRNA expression and NO production between the tumors of L-1-MT treated mice and control mice (Additional file 3: Figure S5A and S5B). The proliferative tumor microvessels promote tumor growth by delivering nutrients and oxygen to tumors. H_2_S has been reported to display anti-angiogenic activity in animal model [25]. Therefore, we immunostained microvessels of tumor sections with endothelial marker CD31 to image the tumor microvasculature (Fig. 6d). Then, according to pathological standards, we measured microvessel parameters: microvessel density, i.e. the number of microvessels in a given area, and percentage coverage, i.e. percentage of microvessels in a given area. The results showed that H_2_S administration significantly reduced microvasculature density (Fig. 6g). Further, the caspase 3 (apoptotic cell marker) level in the tumors from H_2_S administrated mice was higher than that in the control mice (Fig. 6d and f). Taken together, our results indicated that H_2_S administration significantly reduced microvasculature density and induced tumor cell apoptosis, therefore restricted tumor growth in H22 HCC-bearing mice.

### H_2_S exhibited immunotherapeutic efficacy in H22 HCC-bearing mice by inducing T-effector cells and inhibiting MDSCs

**Fig. 7:**
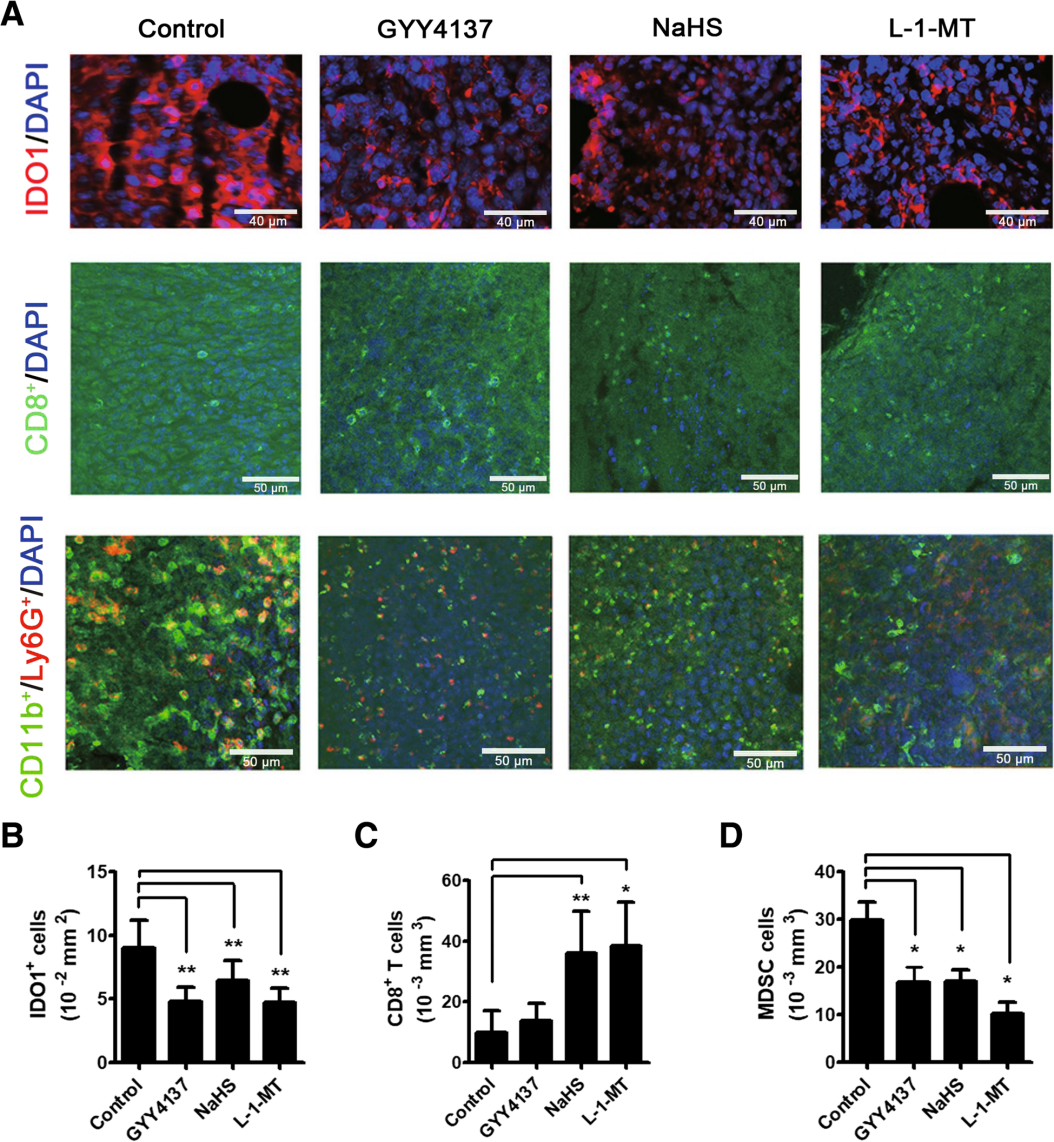
H_2_S exhibited immunotherapeutic efficacy in H22 HCC-bearing mice by inducing T-effector cells and inhibiting MDSCs. **a** Representative immunostaining images of IDO1 protein (× 400 magnification) and CD8^+^ T cells (× 400 magnification) and MDSC cells from each experimental group (× 400 magnification). **b-d** Quantification of IDO1 positive cells, CD8^+^ T cells and MDSC cells in each group. L-1-MT group, *N* = 4, other groups, *N* = 9. Statistical significance was determined by one-way ANOVA followed by Dunnett’s test. *P < 0.05, **P < 0.01, bars show the group mean ± SD

To determine whether the inhibition of tumor growth in H22 HCC-bearing mice caused by H_2_S was related to IDO1 and immune responses, we examined the expression of IDO1 and CD8^+^ T cells in tumor tissues. Consistent with in-vitro results, it was observed that H_2_S downregulated the expression of IDO1 in H22 HCC-bearing mice (Fig. 7a and b). It was also found that the number of CD8^+^ T cells in tumors of H_2_S treated mice was significantly higher than that of control (Fig. 7a and c). It also has been known that the accumulation of MDSC cells inhibits CD8^+^ T cell activation in solid tumor [37]. Mouse MDSCs can be labeled by CD11b and Ly6G expressed by macrophage and granulocyte [37–39]. To find the effect of H_2_S on MDSCs, we evaluated the number of (CD11b^+^/Ly6G^+^) MDSC cells in tumors and found that H_2_S donors reduced the number of MDSCs (Fig. 7a and d). In summary, these results suggested that the restriction of tumor growth by H_2_S was related to the downregulation of IDO1 expression and induction of CD8^+^ T cells and inhibition of MDSCs.

## Discussion

Tumors use multiple suppressive strategies to escape from antitumor immune surveillance and metastasize to target sites. IDO1 has been known as a major mechanism of immunosuppression in tumors. However, the regulation of IDO1 expression and activity is poorly understood. Although gaseous signaling molecule NO has been reported to play an important role in the inhibition and degradation of IDO1 [16–19], the effect of another gaseous signaling molecule H_2_S on IDO1 has never been reported.

Here, using *Cse*^*−/−*^ mice and MCF-7 and SGC-7901 cells, we showed that H_2_S downregulated IDO1 expression via blocking the STAT3 and NF-κB pathways. We also found that H_2_S affected the expression of NO-generating enzyme *iNOS* and the production of NO. H_2_S and NO are known to interact each other, alter the expression of their respective synthesizing enzymes, and also affect each other’s downstream metabolic pathways [27]. It has been reported that NO upregulates the mRNA levels of H_2_S-generating enzyme CSE and increases the production of H_2_S [28]. In addition, H_2_S-generating enzymes are correlated to the enzymes involved in NO production, and trigger the NO-production signaling cascade under shear flow conditions [29]. Whether the regulation of H_2_S on IDO1 expression via STAT3 and NF-κB pathways is dependent on NO still remains to be further investigated, because the effect of NO on INF-γ induced IDO1 expression is controversial [16, 17], although the correlation between NO and STAT3 and NF-κB pathways has been reported [40, 41]. Besides the correlation between H_2_S and IDO1 expression, the relation between H_2_S-generating enzyme CSE and IDO1 expression was also explored in *Cse*^*−/−*^ mice, MCF-7 and SGC-7901 cells and HCC patients. CSE expression was always negatively correlated with IDO1 expression in both tumors and adjacent non-neoplastic tissues of HCC patients. Moreover, the negative correlation between IDO1 expression and CSE expression was further confirmed with the tissue arrays of HCC and MHCC patients. It seemed that the negative correlation between IDO1 and CSE had a certain universality.

Using *Cse*^*−/−*^ mice and MCF-7 and SGC-7901 cells, we showed that H_2_S inhibited the activity of IDO1 by promoting the generation of NO by iNOS. In IDO1 and CSE co-expressing SGC-7901 cells, H_2_S significantly decreased the activity of IDO1 but increased the production of NO. Our data clearly showed that H_2_S inhibited IDO1 activity in a NO-dependent manner. Using the in-vitro enzymatic assay of IDO1, the potent IDO1 inhibitory activity of H_2_S was evaluated for the first time. H_2_S was found to be inactive in IDO1 inhibition, while NO donor SNP was confirmed to be a potent IDO1 inhibitor with IC_50_ value of 1.05 μM. Our finding was consistent with the previous report that NO could inhibit IDO1 activity by forming an IDO1-NO-Trp complex in a reversible manner [17]. In conclusion, H_2_S inhibited IDO1 activity via H_2_S/NO crosstalk. IDO1 has emerged as a new significant therapeutic target due to its indispensable immunomodulatory roles in pregnancy [42], cancer [43], allergy [44] and central nervous system disorders such as Alzheimer’s disease (AD) [45]. Several pharmacological inhibitors of IDO1 are available, and some of them have entered clinical trials [12]. However, the failure of clinical trials of several IDO1 inhibitors including INCB024360, GDC-0919 and F001287 has been known since last winter. Thus, discovery of new IDO1 inhibition strategy has become extremely urgent. Recently, many studies have reported the therapeutic potential of NO, H_2_S and their analogs. For example, NOSH-aspirin (NBS-1120), a NO- and H_2_S-releasing hybrid has potent anti-cancer properties [46]. Therefore, H_2_S, NO and/or their analogs bearing IDO1 inhibition potential can be developed as novel immunotherapeutic drugs.

Using H22 HCC-bearing mice, we found that H_2_S restricted tumor growth and exhibited immunotherapeutic efficacy by downregulating IDO1 expression, inducing T-effector cells, and inhibiting MDSCs. IDO1 recruits tumor-infiltrating MDSCs into tumor milieu and is responsible for MDSC-associated activation and/or recruitment of Tregs in cancer [47]. Herein, we found that CD11b^+^ myeloid cells were enriched in IDO1 high-expressed but not in IDO1 low-expressed tumor area of HCC patients. It has been reported that the treatment efficacy of IL-12 in murine HCC can be limited by the upsurge of IDO1 mediated immunosuppression [48]. Moreover, CTLA-4-dependent IL-10 and IDO1 production are involved in the suppression of CD4^+^ T-cell response by CD14^+^DCs in HCC [49]. Thus, our study provides a previously unreported immunotherapy strategy for HCC using H_2_S targeting IDO1. Our study with H22 HCC-bearing mice showed that H_2_S donors reduced the number of MDSCs, increased the number of CD8^+^ T cells, and downregulated IDO1 expression in tumors. H_2_S donors (DTTs, S-NSAIDs and S-valproate) are demonstrated to bear significant anti-angiogenic activities, through inhibition of endothelial cell proliferation, vascular cell outgrowth and extracellular matrix invasion [50]. In our study, we found that the anti-tumor effects of H_2_S in H22 HCC-bearing mice were associated with its immunomodulatory activity.

## Conclusions

Collectively, our data showed that H_2_S is a promising immunomodulator due to its negative regulation on the immunosuppressive enzyme IDO1 and represents a novel therapeutic target in cancer therapy.

## Additional file

Additional file 1: Supplementary material and methods. (DOCX 32 kb)

Additional file 2: **Table S1.** Primers used for qPCR analysis of gene expression. **Table S2.** Sequences of recombinant vectors used in this study (DOCX 25 kb)

Additional file 3: **Figure S1.** Endogenous H_2_S-generating enzyme CSE negatively regulated IDO1 expression in cancer cells. **Figure S2.** H_2_S increased NO production in cancer cells. **Figure S3.** H_2_S decreased the production of kynurenine and downregulated the transcription of *CYP1A1* and *CYP1B1*. **Figure S4.** Negative correlation between CSE expression and IDO1 expression in tissue arrays of HCC and MHCC patients. **Figure S5.** H_2_S increased *Inos* mRNA expression and NO production in H22 HCC-bearing mice. (DOCX 2729 kb)

## Abbreviations

3-MST: 3-mercaptopyruvate sulfurtransferase
AG490: Tyrphostin B42
AhR: Aromatic hydrocarbon receptor
AhR-ARNT: AhR-Ah receptor nuclear translocator
ANOVA: One-way analysis of variance
CAPE: Caffeic acid phenethyl ester
CBS: Cystathionine β-synthase
CSE: Cystathionine γ-lyase
CYP1A1: Cytochrome P4501A1
CYP1B1: Cytochrome P4501B1
H_2_S: Hydrogen sulfide
HCC: Hepatocellular carcinoma
IDO1: Indoleamine 2, 3 -dioxygenase 1
IFN-γ: IFN-gamma
IL-6: Interleukin-6
iNOS: inducible nitric oxide synthase
KP: Kynurenine pathway
Kyn: Kynurenine
L-1-MT: L-1-methyl-tryptophan
LPS: Lipopolysaccharide
MDSCs: Myeloid-derived suppressor cells
MHCC: metastatic hepatocellular carcinoma
NO: Nitric oxide
PPG: DL-propargylglycine
SNP: Sodium nitroprusside
SOCS3: Suppressor of cytokine signaling 3
Teff: Effector T cells
Treg: Regulatory T cells
TRP: Tryptophan
