# Supplementary material for: H2S suppresses indoleamine 2, 3-dioxygenase 1 and exhibits immunotherapeutic efficacy in murine hepatocellular carcinoma
Source: J Exp Clin Cancer Res. 2019 Feb 18;38:88. doi: 10.1186/s13046-019-1083-5 (PMC6380069; doi:10.1186/s13046-019-1083-5)
Supplement: Supplementary file 1 — Supplementary material and methods. (DOCX 32 kb) [file 13046_2019_1083_MOESM1_ESM.docx]

**Additional File 1**

**H_2_S Suppresses indoleamine 2, 3-dioxygenase 1 and Exhibits Immunotherapeutic** **Efficacy in** **Murine Hepatocellular Carcinoma**

Dan Yang^1^, Tianqi Li^1^, Yinlong Li^1^, Shengnan Zhang^1^, Weirui Li^1^, Heng Liang^1^, Zikang Xing^1^, Lisha Du^1^, Jinchao He^1^, Chunxiang Kuang^2^, Qing Yang^1^*

^1^ State Key Laboratory of Genetic Engineering, Department of Biochemistry, School of Life Sciences, Fudan University, Songhu Road 2005, Shanghai, 200438, China

^2^ Department of Chemistry, Tongji University, Siping Road 1239, Shanghai, 200092, China

*Corresponding author:

Qing Yang, address: State Key Laboratory of Genetic Engineering, Department of Biochemistry, School of Life Sciences, Fudan University, Songhu Road 2005, Shanghai, 200438, China, telephone & fax number: +86-021-31246641, E-mail: yangqing68@fudan.edu.cn.

Author email address:

Dan Yang: 16110700083@fudan.edu.cn

Tianqi Li: 16210700110@fudan.edu.cn

Yinlong Li: 14210700074@fudan.edu.cn

Shengnan Zhang: 17110700084@fudan.edu.cn

Weirui Li: 17210700070@fudan.edu.cn

Heng Liang: 17210700057@fudan.edu.cn

Zikang Xing: 17110700017@fudan.edu.cn

Lisha Du: 14110700103@fudan.edu.cn

Jinchao He: dd_nini@163.com

Chunxiang Kuang: kuangcx@tongji.edu.cn

**Supplementary material and methods**

**Cell culture and transfection.**

MCF-7, SGC-7901 and H22 cells were authenticated by short tandem repeat analysis and passaged for fewer than 6 months before experiments. All cell lines were tested to be negative for mycoplasma contamination. The cells were cultured in DMEM (Gibco, USA) supplement with 10% (vol/vol) fetal bovine serum (FBS, Gibco, USA), 1% (vol/vol) nonessential amino acid solution (NEAA, Gibco, USA), 100 μg/mL penicillin and streptomycin (Gibco, USA) at 37°C, in an atmosphere of 5% CO_2_ and 90% relative humidity.

Cells were seeded in a 6-well culture dish and transfected at approximately 70% confluence. Transfection was performed with Opti-MEM reduced serum medium (Invitrogen, USA), using LipofectamineTM2000 transfection reagent, according to the manufacturer's instructions (Invitrogen, USA). At 4-6 h after transfection, the Opti-MEM reduced serum medium was removed and the cells were cultured in medium supplemented with 10% FBS.

**Animal model and treatments.**

H22 hepatocellular carcinoma (HCC) cells were injected s.c. into the right forelimb at 2×10^6^ of each mouse, respectively. After the implantation, the mice were randomized into several groups: control, L-1-MT, NaHS, and GYY4137. Tumor growth was monitored every day. Perpendicular diameters of the tumors were measured using vernier scale calipers, and the tumor volume was calculated as follows: tumor size = long diameter × (short diameter)^2^/2. When the primary tumor size had reached 100-200 mm^3^, therapy was initiated. The mice were intratumor injected once daily with either control (saline solution), NaHS and GYY4137 and L-1-MT 100 mg/m^2^/day tumor size respectively. After 5 days of therapy, the mice were sacrificed, the tumors were dissected and weighed. All experimental procedures were approved by the Animal Ethics Committee of Fudan University, experiments were performed in compliance with the ARRIVE guidelines.

**Western blot and the primary antibodies.**

Mice kidney, lung, brain, intestine, heart and liver tissues were snap frozen in liquid nitrogen after dissection and stored under -80℃. Before being lysed, 100 mg of tissue samples were ground in liquid nitrogen in a mortar and powders were poured with liquid nitrogen into an Eppendorf tube, and 200 μL of RIPA lysis buffer (50 mM Tris-HCl, pH 7.4, 150 mM NaCl, 1% Triton X-100, 1% sodium deoxycholate, 0.1% SDS, Beyotime, China) containing 5 mM EDTA (Beyotime, China), 100 mM PMSF and Na_3_VO_4_ (Beyotime, China) was supplemented. Cultured cells were collected and incubated in lysis buffer as above. Mouse tissue lysates and cell lysates were kept on ice for 30 min, vortexed, disrupted by a 30 s burst of super sonication, and centrifuged at 13,000 rpm for 3-5 min at 4℃. Supernatants were collected and protein levels were quantified using BCA Protein Assay Kit (Beyotime, China) following the manufacturer's guidelines. Supernatants were boiled in 5× SDS loading buffer, the same amounts of protein were separated by 10% SDS-PAGE and blotted onto polyvinylidene fluoride membranes (Millipore, ISEQ00010, USA), all primary antibody incubations were performed at 4℃ overnight.

The primary antibodies used in the experiment were as follows: IDO1 rabbit PcAb (ab106134, Abcam, USA) and CSE mouse mAb (CTH, sc-374249, Santacruz, USA); phospho-NF-κB rabbit mAb (Ser536, #3033, CST, USA), NF-κB rabbit PcAb (ER0815, HangZhou HuaAn Biotechnology Co., Ltd, China); phospho-STAT3 rabbit PcAb (Tyr705, CY6566, Abways, China), STAT3 rabbit PcAb (CY5292, Abways, China); Phospho-IKK-α (Ser176)/IKK-β (Ser177) rabbit PcAb (CY6290, Abways, China); IKK-α/IKK-β rabbit PcAb (CY3048, Abways, China); Phospho-IκB-α (Ser32/Ser36) rabbit PcAb (CY6280, Abways, China); IκB-α (N-term) mouse mAb (AB3178, Abways, China); blotting for β-Actin using a rabbit mAb (ET1701-80, HangZhou HuaAn Biotechnology Co., Ltd, China) and GAPDH using a mouse mAb (EM1101, HangZhou HuaAn Biotechnology Co., Ltd, China) of corresponding specificity served as the loading control.

**Immunostaining.**

Fresh mouse and human tumor samples were fixed in 4% paraformaldehyde (PFA), paraffin-embedded and cut into 4-6 μm slides. All slides and tissue arrays were dehydrated in gradient ethanol. Following the antigen retrieval done in 10 mM citrate buffer pH 6.0 (Na_3_H_6_H_5_O_7_, Beyotime, China), blocking was done using PBS with 10% normal donkey serum (Sigma-Aldrich, USA) for 40 min. Primary antibodies were incubated overnight at 4°C. The following primary antibodies were used to perform staining: IDO1 (human IDO1, CST, 12006, USA); CSE (CTH, Santacruz, sc-374249, USA); IDO1 (mIDO1-48, Biolegend, 122402, USA). The following secondary antibodies were used: AlexaFluor 488 donkey anti-mouse (Invitrogen, USA), AlexaFluor 555 donkey-anti-rabbit (Invitrogen, USA), AlexaFluor 488 donkey-anti-rat (Invitrogen, USA) and DAPI (Sigma-Aldrich, USA). Secondary antibodies were left for 1 h at room temperature. The immunostaining analysis was carried out under a laser scanning confocal microscope (Carl Zeiss AG, LSM710, Germany).

Fresh mouse and human tumor samples were fixed in 4% PFA, embedded in optimum cutting temperature (OCT) compound and cut in thick tissue sections (45µm). Triton X-100 (0.2%) was used to permeabilize cells and blocking was done using with 10% normal goat serum or normal donkey serum (Sigma-Aldrich, USA). Primary antibodies were incubated overnight at 4°C and secondary antibodies were incubated for 1 h at room temperature. The following primary antibodies were used: IDO1 (human IDO1, CST, 12006, USA); CSE (CTH, Santacruz, sc-374249, USA); E-cadherin (R&D, AF748, USA); Caspase 3 (CST, 9579, USA); CD31 (BD, 553370, USA); CD8 (Abcam, ab203035, USA); CD11b (Abcam, ab133357, USA); Ly6G (Abcam, ab25377, USA). The following secondary antibodies were used: AlexaFluor 488 donkey anti-rabbit (Invitrogen, USA), AlexaFluor 555 donkey-anti-rabbit (Invitrogen, USA), AlexaFluor 647 donkey-anti-goat (Invitrogen, USA), AlexaFluor 555 donkey-anti-rat (Invitrogen, USA) and DAPI (Sigma-Aldrich, USA). The 4 X 4 large Z-stack scanned images were performed in Nikon imaging software (Nikon, A1-Ni, Japan).

**H&E staining.**

For histology analysis, excised tumors were fixed in 4% PFA and processed for routine hematoxylin and eosin staining (Hematoxylin and Eosin Staining Kit, Beyotime, China).

**Detection of NO production.**

Total NO production was measured by a Nitrate/Nitrite Assay Kit (Beyotime, China) following the manufacturer's guidelines. Briefly, 60 μL cell samples were mixed with 5 μL of NADPH, 10 μL of FAD and 5 μL of nitrate reductase and incubated at 37℃ for 30 min to fulfill the conversion of nitrate to nitrite. To the above mixture, 10 μL of LDH solution was added and incubated at 37℃ for 30 min to eliminate the redundant NAPDH. Then, 50 μL of the Griess reagent I and Griess reagent II were added and incubated at 37℃ for another 30 min. The mixture was left for 10 min at room temperature and the absorbance at 540 nm was measured on ELx800 microtiter plate reader (BioTek, USA). Concentrations were determined by comparison with a sodium nitrite standard curve.

**High-performance liquid chromatography (HPLC) analysis.**

The IDO1 activities of mouse serum and cell supernatant were evaluated by measuring the concentrations of Trp and Kyn using an Agilent 1260 series HPLC system (Agilent Corp., USA) equipped with a quad pump and a UV detector. The detection wavelengths were 280 nm and 360 nm. HPLC analysis of the samples was performed using an Agilent C18 column (5 μm particle size, L × I.D. 25 cm × 4.6 mm) preceded by a C18 guard column (Dikma, China). The mobile phase was 15 mM sodium acetate (pH 3.6) containing 4% acetonitrile.

**Analyzing the correlations between IDO1 expression and CSE expression,** **CD11b^+^ myeloid cell number or CD8^+^ T cell number.**

We quantified the immunofluorescence intensity of IDO1 or CSE expression in each HCC patient sample and defined the high IDO1 or CSE expression sample as the one whose immunofluorescence intensity value of IDO1 or CSE was higher than the median of all samples. We also defined the low IDO1 or CSE expression sample as the one whose immunofluorescence intensity value of IDO1 or CSE was lower than the median of all samples. In the same way, samples that have a large number of CD11b+ myeloid cells and CD8^+^ T cells and samples that have a small number of these two kinds of cells were differentiated. The scatter gram was plotted using Prism 6 software (GraphPad Software). IDO1 expression was placed on the x-axis and CSE expression, CD11b+ myeloid cell number or CD8^+^ T cell number was respectively placed on the y-axis. For visualization purpose, a linear model based on the data was constructed.

Heatmaps analysis of the correlations between IDO1 expression and CSE expression, CD11b+ myeloid cell number or CD8^+^ T cell number were performed by R statistical software. Tumor samples of 158 HCC patients were immunostained, and 126 valid immunostaining results were collected and analyzed. The rest of the results were neglected because of the lost or weak immunostaining. For consistency, tumor samples of 158 MHCC patients were immunostained and a similar number of samples which is 124 were collected from line C to line K after removing the invalid results. These exclusion criteria were pre-established. Tested values were normalized used by the function scale (), and heatmaps were plotted using pheatmap R package. The function cor.test (x, y, method = “spearman”) was used to calculate the Spearman correlation coefficient and *p* value that represent the correlations between IDO1 expression and CSE expression, CD11b+ myeloid cell number or CD8^+^T cell number.
