# Supplementary material for: H2S suppresses indoleamine 2, 3-dioxygenase 1 and exhibits immunotherapeutic efficacy in murine hepatocellular carcinoma
Source: J Exp Clin Cancer Res. 2019 Feb 18;38:88. doi: 10.1186/s13046-019-1083-5 (PMC6380069; doi:10.1186/s13046-019-1083-5)
Supplement: Supplementary file 2 — Table S1. Primers used for qPCR analysis of gene expression. Table S2. Sequences of recombinant vectors used in this study (DOCX 25 kb) [file 13046_2019_1083_MOESM2_ESM.docx]

**Additional File 2**

**H_2_S Suppresses indoleamine 2, 3-dioxygenase 1 and Exhibits Immunotherapeutic** **Efficacy** **in** **Murine Hepatocellular Carcinoma**

Dan Yang^1^, Tianqi Li^1^, Yinlong Li^1^, Shengnan Zhang^1^, Weirui Li^1^, Heng Liang^1^, Zikang Xing^1^, Lisha Du^1^, Jinchao He^1^, Chunxiang Kuang^2^, Qing Yang^1^*

^1^ State Key Laboratory of Genetic Engineering, Department of Biochemistry, School of Life Sciences, Fudan University, Songhu Road 2005, Shanghai, 200438, China

^2^ Department of Chemistry, Tongji University, Siping Road 1239, Shanghai, 200092, China

*Corresponding author:

Qing Yang, address: State Key Laboratory of Genetic Engineering, Department of Biochemistry, School of Life Sciences, Fudan University, Songhu Road 2005, Shanghai, 200438, China, telephone & fax number: +86-021-31246641, E-mail: yangqing68@fudan.edu.cn.

Author email address:

Dan Yang: 16110700083@fudan.edu.cn

Tianqi Li: 16210700110@fudan.edu.cn

Yinlong Li: 14210700074@fudan.edu.cn

Shengnan Zhang: 17110700084@fudan.edu.cn

Weirui Li: 17210700070@fudan.edu.cn

Heng Liang: 17210700057@fudan.edu.cn

Zikang Xing: 17110700017@fudan.edu.cn

Lisha Du: 14110700103@fudan.edu.cn

Jinchao He: dd_nini@163.com

Chunxiang Kuang: kuangcx@tongji.edu.cn

**Supplementary** tables

**T**able S1. Primers used for qPCR analysis of gene expression.

| **Target Gene** | **Primer sequence (5'-3')** |
| --- | --- |
| Human IDO1-F (qPCR) | ATGCAAGAACGGGACACT |
| Human IDO1-R (qPCR) | GCCTTTCCAGCCAGACAA |
| Human iNOS-F (qPCR) | TCGGGCTGAAGTGGTATG |
| Human iNOS-R (qPCR) | AGGTCTCGGACTCCAATCT |
| Human CYP1A1-F (qPCR) | CTCCTCAACCTCCTGCTAC |
| Human CYP1A1-R (qPCR) | GTGGTTGTGGTCATAGCG |
| Human CYP1B1-F (qPCR) | CGCTCCTGCTACTCCTGTCG |
| Human CYP1B1-R (qPCR) | TCGCCATTCAGCACCACTA |
| Human β-Actin-F (qPCR) | CGGGAAATCGTGCGTGAC |
| Human β-Actin-R (qPCR) | GGAAGGAAGGCTGGAAGAG |
| Mouse Ido1-F (qPCR) | ATCCTTGAAGACCACCAC |
| Mouse Ido1-R (qPCR) | AGGCAGATTTCTAGCCAC |
| Mouse Inos-F (qPCR) | GAGCGAGTTGTGGATTGTC |
| Mouse Inos-R (qPCR) | CCAGGAAGTAGGTGAGGG |
| Mouse β-Actin-F (qPCR) | CTGTCCCTGTATGCCTCTG |
| Mouse β-Actin-R (qPCR) | ATGTCACGCACGATTTCC |

**T**able S2. **Sequences of recombinant vectors used in this study.**

| **Target Gene** | **Primer sequence (5'-3')** |
| --- | --- |
| pcDNA-3.1(+)-CSE-F | TCCACTAGTCCAGTGTGGTGGAATTCGCCACCAT |
|  | GCAGGAAAAAGACGCCTCCTC |
| pcDNA-3.1(+)-CSE-R | GTTTAAACGGGCCCTCTAGACTCGAGCTAGCTGT |
|  | GACTTCCACTTGGAGGG |
| px459-cas9-CSE-F | CACCGCTTTCGCGGTTCAGCATGC |
| px459-cas9-CSE-R | AAACGCATGCTGAACCGCGAAAGC |
| pGL3-Promoter-DRE-F | ATTTCTCTATCGATAGGTACCTCGCGTGTCGCGTG |
|  | TCGCGTGTCGCGTGTCGCGTGTCGCGTGAGATCT |
|  | GCGATCTAAG |
| pGL3-Promoter-DRE-R | CTTAGATCGCAGATCTCACGCGACACGCGACACG |
|  | CGACACGCGACACGCGACACGCGAGGTACCTATC |
|  | GATAGAGAAAT |
