# Supplementary material for: H2S suppresses indoleamine 2, 3-dioxygenase 1 and exhibits immunotherapeutic efficacy in murine hepatocellular carcinoma
Source: J Exp Clin Cancer Res. 2019 Feb 18;38:88. doi: 10.1186/s13046-019-1083-5 (PMC6380069; doi:10.1186/s13046-019-1083-5)
Supplement: Supplementary file 3 — Figure S1. Endogenous H2S-generating enzyme CSE negatively regulated IDO1 expression in cancer cells. Figure S2. H2S increased NO production in cancer cells. Figure S3. H2S decreased the production of kynurenine and downregulated the transcription of CYP1A1 and CYP1B1. Figure S4. Negative correlation between CSE expression and IDO1 expression in tissue arrays of HCC and MHCC patients. Figure S5. H2S increased Inos mRNA expression and NO production in H22 HCC-bearing mice. (DOCX 2729 kb) [file 13046_2019_1083_MOESM3_ESM.docx]

**Additional File 3**

**H_2_S Suppresses indoleamine 2, 3-dioxygenase 1 and Exhibits Immunotherapeutic** **Efficacy** **in** **Murine Hepatocellular Carcinoma**

Dan Yang^1^, Tianqi Li^1^, Yinlong Li^1^, Shengnan Zhang^1^, Weirui Li^1^, Heng Liang^1^, Zikang Xing^1^, Lisha Du^1^, Jinchao He^1^, Chunxiang Kuang^2^, Qing Yang^1^*

^1^ State Key Laboratory of Genetic Engineering, Department of Biochemistry, School of Life Sciences, Fudan University, Songhu Road 2005, Shanghai, 200438, China

^2^ Department of Chemistry, Tongji University, Siping Road 1239, Shanghai, 200092, China

*Corresponding author:

Qing Yang, address: State Key Laboratory of Genetic Engineering, Department of Biochemistry, School of Life Sciences, Fudan University, Songhu Road 2005, Shanghai, 200438, China, telephone & fax number: +86-021-31246641, E-mail: yangqing68@fudan.edu.cn.

Author email address:

Dan Yang: 16110700083@fudan.edu.cn

Tianqi Li: 16210700110@fudan.edu.cn

Yinlong Li: 14210700074@fudan.edu.cn

Shengnan Zhang: 17110700084@fudan.edu.cn

Weirui Li: 17210700070@fudan.edu.cn

Heng Liang: 17210700057@fudan.edu.cn

Zikang Xing: 17110700017@fudan.edu.cn

Lisha Du: 14110700103@fudan.edu.cn

Jinchao He: dd_nini@163.com

Chunxiang Kuang: kuangcx@tongji.edu.cn

**Supplementary figures and figure legends**

Figure S1.


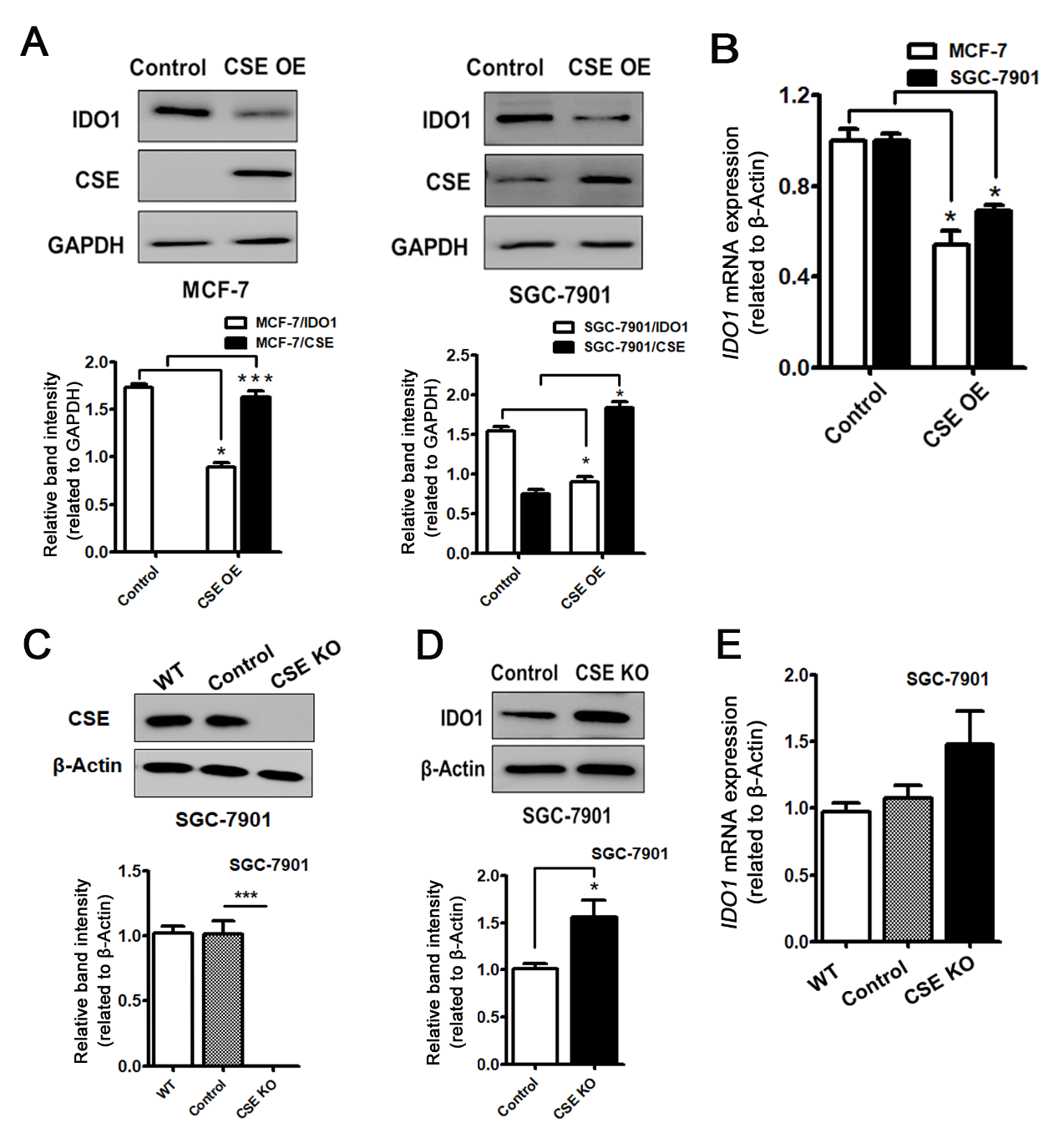
 **Figure** S1 **(related to Figure 2).** **Endogenous H_2_S-generating enzyme CSE negatively regulated IDO1 expression in cancer cells.**

**(A, B)** IDO1 expression was decreased in CSE OE cells. The CSE OE (CSE-overexpressing) MCF-7 and SGC-7901 cells were constructed, total RNA and protein were extracted and IDO1 expression was quantitatively analyzed by qPCR and western blot. **(C-E)** IDO1 expression was increased in CSE KO SGC-7901 cells. The CSE KO (CSE knockout) SGC-7901 cells were constructed, total RNA and protein were extracted and IDO1 expression was quantitatively analyzed by qPCR and western blot. All experiments repeated at least three times. Student's t test, **P*< 0.05, ***P*< 0.01, bars show the group mean ± SEM.

**Figure** S2.


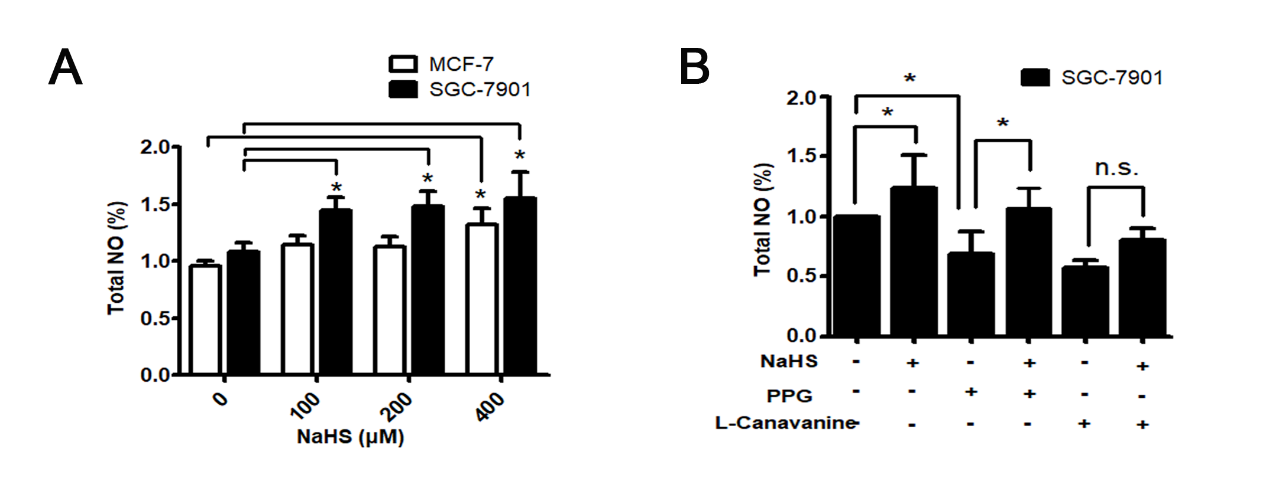
 **Figure** S2 **(related to Figure 3).** **H_2_S increased NO production in cancer cells.**

**(A)** MCF-7 and SGC-7901 cells were cultured overnight after seeding in a six-well plate and were then treated with NaHS at different concentrations for 24 h. NO production was measured by a Nitrate/Nitrite Assay Kit. **(B)** SGC-7901 cells were treated with NaHS (H_2_S donor, 400 μM) for 1 h, PPG (DL-propargylglycine, CSE inhibitor, 10 mM) for 4 h or L-canavanine (iNOS inhibitor, 200 μM) for 2 h. NO production was measured by a Nitrate/Nitrite Assay Kit. Data were obtained from three independent experiments. Statistical significance was determined by one-way analysis of variance (ANOVA) followed by Dunnett’s test. **P*< 0.05, ***P*< 0.01, bars show the group mean ± SEM.

**Figure** S3.


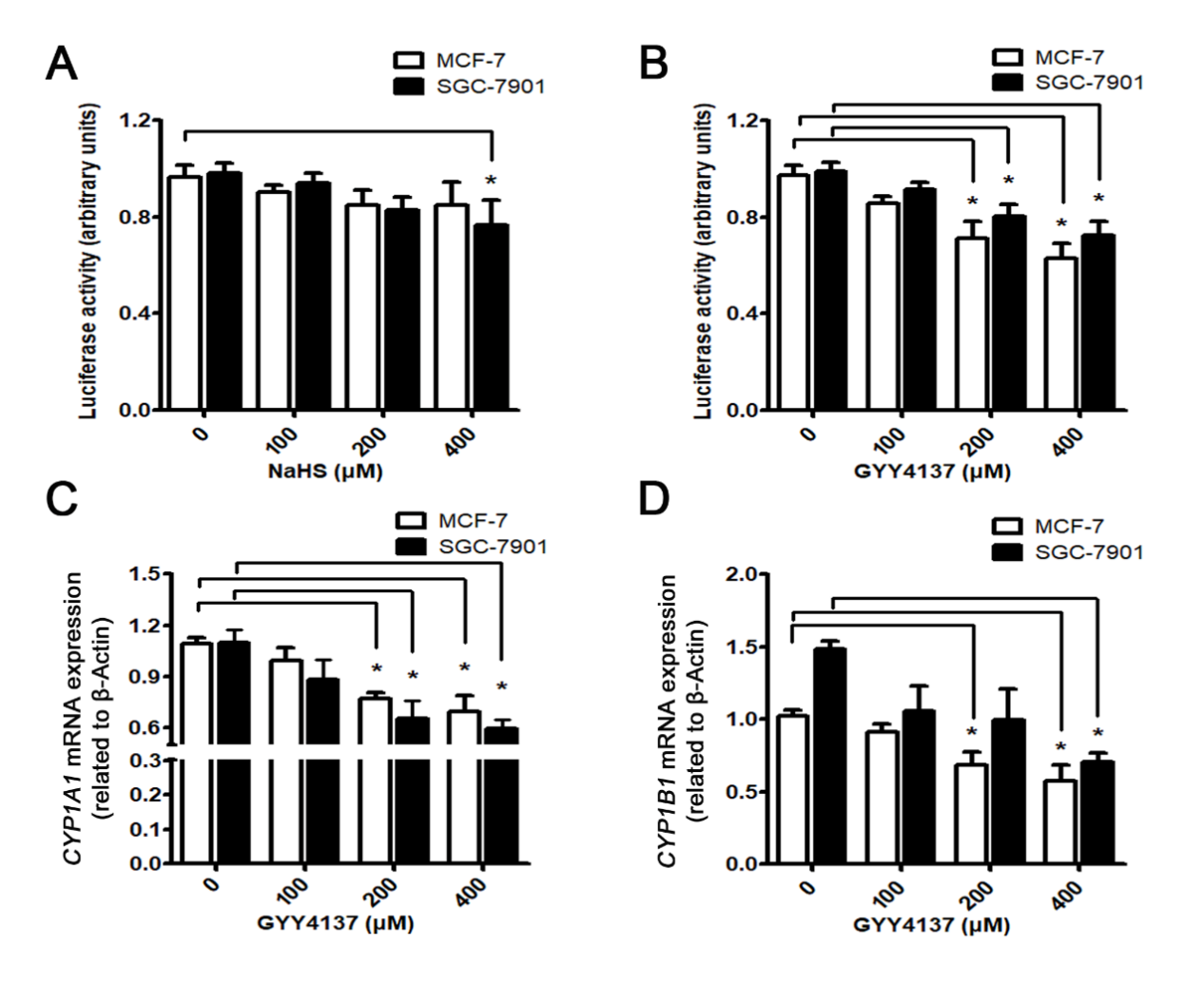
 **Figure** S3 **(related to Figure 3).** **H_2_S decreased the production of kynurenine and downregulated the transcription of *CYP1A1* and *CYP1B1*.**

**(A, B)** Luciferase reporter assay of kynurenine, a tryptophan metabolite. MCF-7 and SGC-7901 cells were transfected with pGL3-Promoter vector-DRE and a pSV-β-Galactosidase control vector then incubated with NaHS or GYY4137 at different concentrations (0-400 μM) for 24 h. The luciferase activity of treated cells was analyzed by luciferase assays. **(C, D)** The mRNA expression levels of AhR-ARNT target genes *CYP1A1* and *CYP1B1*. Total RNA was extracted from the SGC-7901 or MCF-7 cells that were treated with different concentrations of GYY4137 (0-400 μM) for 24 h, and mRNA expression of *CYP1A1* and *CYP1B1* was analyzed by qPCR. Data were obtained from three independent experiments. Statistical significance was determined by one-way analysis of variance (ANOVA) followed by Dunnett’s test. **P*< 0.05, bars show the group mean ± SEM.

**Figure** S4.


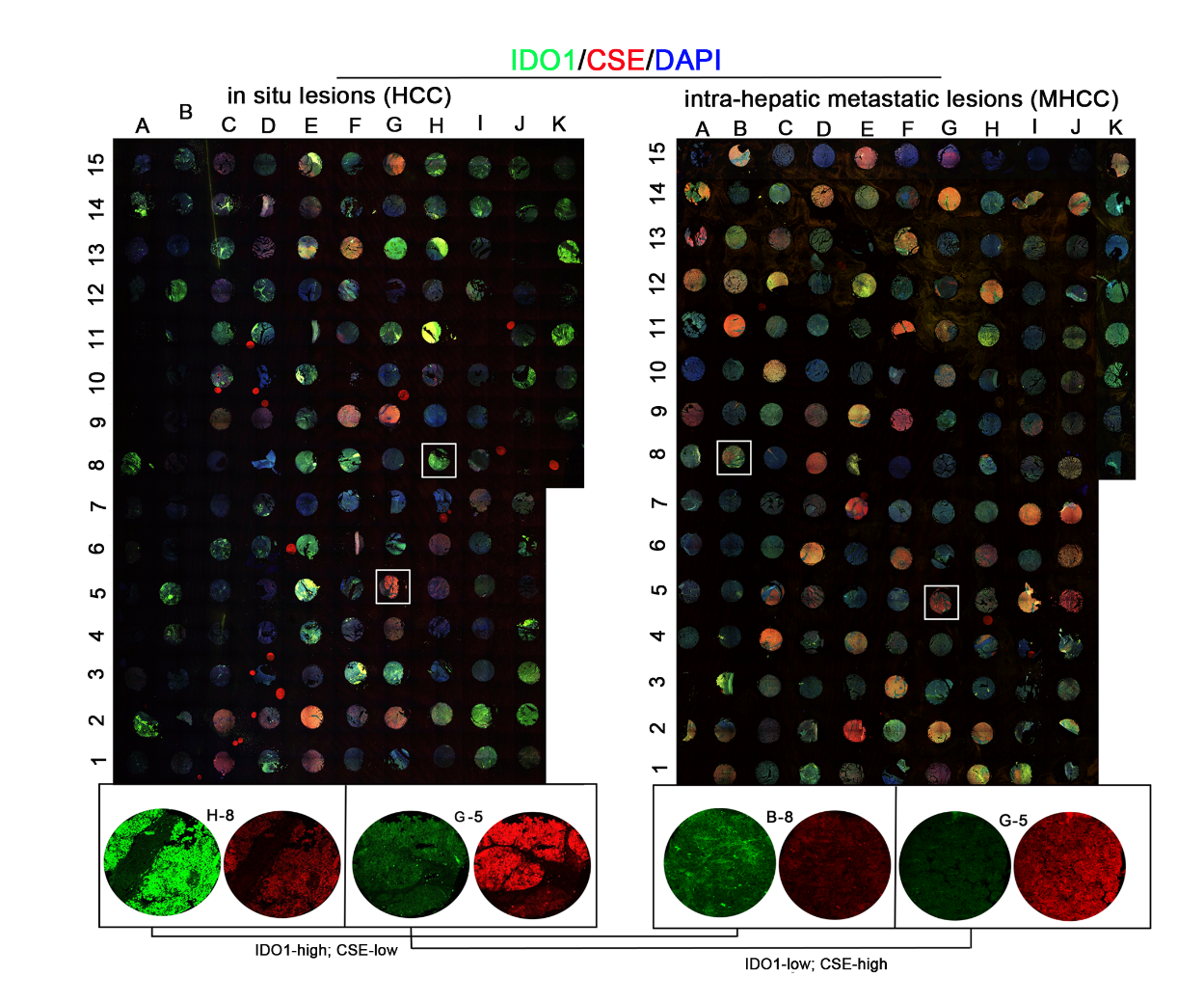
 **Figure** S4 **(related to Figure 5).** **Negative correlation between CSE expression and IDO1 expression in tissue arrays of HCC and MHCC patients.**

Immunostaining of IDO1 and CSE in the tumors of HCC (158 cases) and metastatic hepatocellular carcinoma MHCC (158 cases) patients.

**Figure** S5.


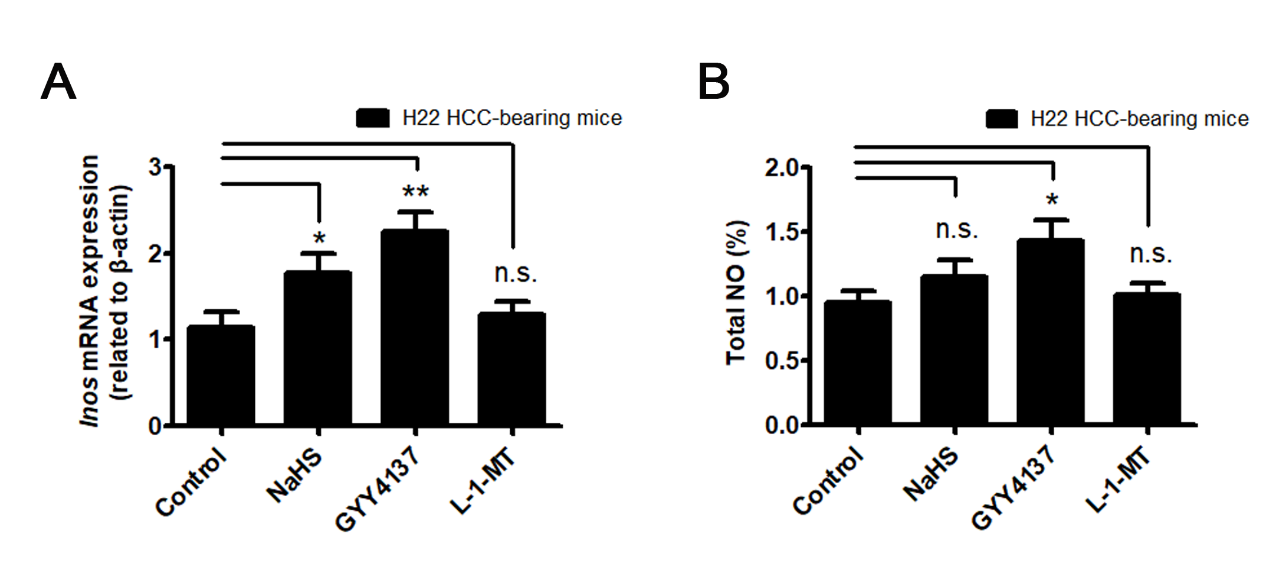
 **Figure S5 (related to Figure 6). H_2_S increased *Inos* mRNA expression and NO production in H22 HCC-bearing mice.**

(A) Total RNA was extracted from the tumors of different groups of mice. *Inos* mRNA expression was detected by qPCR. (B) Total protein was extracted from the tumors of different groups of mice. NO production was measured by a Nitrate/Nitrite Assay Kit. Data were obtained from three independent experiments. L-1-MT group, N=4, other groups, N=9. Statistical significance was determined by one-way analysis of variance (ANOVA) followed by Dunnett’s test. **P*< 0.05, ***P*< 0.01, n.s., no signiﬁcant difference, bars show the group mean ± SEM.
